# Low‐Dose‐Rate Prostate Brachytherapy (LDR‐PB) adopts postsurgical PSA value for definition of cure

**DOI:** 10.1002/bco2.49

**Published:** 2020-10-19

**Authors:** Jennifer Uribe, Santiago Uribe‐Lewis, Sara Khaksar, Carla Perna, Christos Mikropoulos, Sophie Otter, Robert Laing, Stephen Langley

**Affiliations:** ^1^ The Stokes Centre for Urology Royal Surrey Hospital NHS Foundation Trust Guildford UK

Long‐term remission, and likely cure, of clinically localized prostate cancer is considered when there is no evidence of prostate‐specific antigen (PSA) or radiographic progression 10 years after initial localized therapy.^1^ The PSA level thresholds to define the posttreatment (biochemical) progression differ based on the mechanism of action of a surgical or radiotherapeutic approach, making cross‐modality comparisons cumbersome. A PSA threshold of ≥0.2 ng/mL is commonly used to report biochemical relapse after radical prostatectomy (RP). In contrast, radiation oncologists most often use the Phoenix definition, a threshold 10‐fold greater, of 2 ng/mL above the posttreatment nadir when reporting the outcome of external beam radiotherapy (EBRT) or brachytherapy. Permanent implants with low‐dose‐rate brachytherapy seeds (LDR‐PB) have shown over the last 20 years to be a very effective ablative treatment for early prostate cancer resulting in low and stable PSA values.

The recent large collaborative study by Crook et al identified a PSA threshold value after LDR‐PB associated with cure, defined as long‐term (10‐15 year) freedom from prostate cancer.^2^ Prospective data were collected from 7 institutions identifying 14 220 patients with localized prostate cancer who were treated with LDR‐PB, either alone (n = 8552) or in combination with EBRT (n = 1175), androgen deprivation (n = 3165), or both (n = 1328). Of the 14 220 cases 8746 patients were selected based on absence of clinical failure 4 years (±6 months) after treatment and availability of a 4‐year PSA value. The median (range) follow‐up time was 8 (3.5‐21.3) years. For patients with 4‐year PSA ≤ 0.2 ng/mL, the threshold for RP, the freedom‐from‐recurrence (FFR) rates were 98.7% at 10 years and 96.1% at 15 years.

These results were validated using three independent prospective prostate brachytherapy data sets. One was the brachytherapy arm of the ASCENDE‐RT phase III randomized trial. The trial compared biochemical progression‐free survival (bPFS) in unfavorable intermediate (31%) and high‐risk patients (69%) who underwent dose escalated EBRT plus ADT, with or without an LDR‐PB boost, with more than 10 years follow‐up. The published results of the trial showed that men randomized to the LDR‐PB boost were twice as likely to be free of biochemical failure at a median follow‐up of 6.5 years.^3^

Interestingly the authors re‐analyzed ASCENDE‐RT data using a surgical PSA threshold of ≤0.2 ng/mL.^4^ In the trial, all subjects had undetectable PSA values at the completion of radiation therapy/ADT, thus providing a unique opportunity to evaluate bPFS after definitive radiation therapy using the surgical threshold to define biochemical relapse. Compared to the nadir + 2 ng/mL definition, the >0.2 ng/mL PSA threshold doubled the number of relapse events from 69 to 139. However, this increase was confined to the DE‐EBRT arm of the trial. The 7‐year Kaplan‐Meier bPFS after DE‐EBRT declined from 76% using nadir + 2 ng/mL to 38% using the >0.2 ng/mL threshold (*P* < .001). Among the LDR‐PB subset, there was no significant difference, the 7‐year Kaplan‐Meier bPFS was 85% (>0.2 ng/mL) vs 88% (nadir + 2 ng/mL) (*P* = .319). The authors concluded that combined modality therapy using a brachytherapy boost provided bPFS outcomes for men with unfavorable risk disease that are at least as good as any published results for RP.

In view of the work reported by Crook et al,^2^ we analyzed the 10‐ and 15‐year FFR rates from our own prostate brachytherapy database that currently holds baseline, treatment, and prospective follow‐up details of over 4500 patients. Clinical relapse in our series was defined as local, nodal, or distant recurrence, or biochemical failure triggering salvage treatment. After selection for patients without relapse within 3.5 years from implantation and an available PSA measurement 48 months (±6 months) post‐implant we identified 1923 cases. The median (range) follow‐up time was 10 years (4‐21) and age at treatment 65 years (44‐83). There were 681 (35%) low‐risk patients, 997 (52%) intermediate, and 245 (13%) high‐risk. Treatment types were LDR‐PB monotherapy in 1279 (67%) patients, ADT + LDR‐PB in 385 (20%), and ADT + EBRT + LDR‐PB boost in 259 (13%). Kaplan‐Meier analyses showed overall 10‐ and 15‐year FFR rates of 98% and 95% in patients with a 48‐month PSA ≤ 0.2 ng/mL (Figure 1); multivariable Cox proportional hazards regression,^5^ adjusted for age at treatment, risk classification, and treatment modalities (including the use of ADT), indicated survival rates were not dependent on these predictor variables in patients with a 48‐month PSA ≤ 0.2 ng/mL.

**FIGURE 1:**
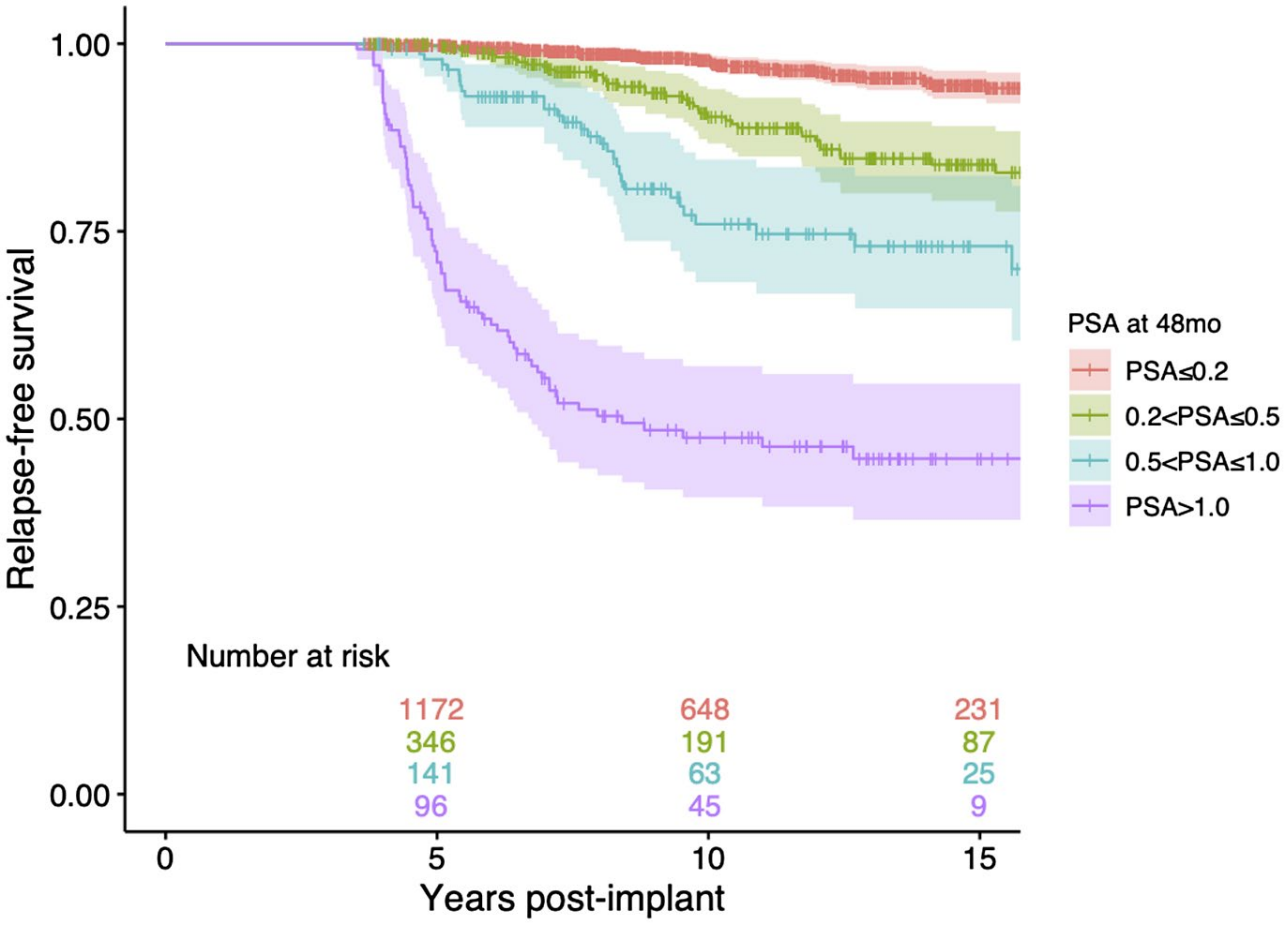
Kaplan‐Meier relapse‐free survival analysis of patients who received LDR‐PB (alone or as combination with ADT and/or EBRT) stratified by their PSA value (ng/mL) 48 months post‐implant

Previously we reported that 92% of relapse‐free high‐risk patients with an available PSA 10 years after implantation had a PSA level ≤ 0.2 ng/mL.^6^ Therefore, from our own long‐term experience and that of others, adoption of a PSA value of ≤ 0.2 ng/mL at 48 months posttreatment as a biochemical definition of cure for patients treated with LDR‐PB is reasonable and evidence based. Importantly, a 48‐month PSA value > 0.2 ng/mL does not necessarily translate into a longer term clinical relapse but does mean such patients would need continued surveillance and reassuringly many of which may ultimately be deemed rid of their disease.

## CONFLICT OF INTEREST

JU and SUL report personal fees from BXTAccelyon Limited and Theragenics Corporation, outside the submitted work. SL and RL report personal fees, nonfinancial support, and other from BXTAccelyon Limited, outside the submitted work. SK, CP, CM, and SO have nothing to disclose. All authors have read and approved the final article.
